# Identification of characteristic genes in cutaneous squamous cell carcinoma based on weighted gene co-expression network analysis

**DOI:** 10.3389/fgene.2025.1470584

**Published:** 2025-04-14

**Authors:** Qipeng Xiao, Pengfei Xu, Wenjun Xu, Qiuhe Song, Yousheng Mao

**Affiliations:** ^1^ Department of Dermatology, Affiliated Hospital of Jiujiang University, Jiujiang, Jiangxi, China; ^2^ College of Nursing, Jiujiang Vocational University, Jiujiang, Jiangxi, China; ^3^ Jiujiang Clinical Precision Medicine Research Center, Affiliated Hospital of Jiujiang University, Jiujiang, Jiangxi, China

**Keywords:** cutaneous squamous cell carcinoma, weighted gene co -expression network analysis, characteristic genes, immune microenvironment (IME), LASSO

## Abstract

**Objective:**

This study aims to identify characteristic genes associated with cutaneous squamous cell carcinoma (cSCC).

**Methods:**

Differentially expressed genes (DEGs) and hub genes in key module were identified using the limma package and weighted gene co-expression network analysis (WGCNA) in R software, respectively. The intersection of these genes was then subjected to LASSO regression to pinpoint characteristic genes. The correlation between immune cell infiltration and these characteristic genes was further elucidated using single-sample Gene Set Enrichment Analysis and Spearman correlation analysis.

**Results:**

A total of 113 DEGs were identified, along with their associated biological pathways. From this pool, five characteristic genes—*ADH1B*, *CCL27*, *ID4*, *LRP4* and *S100A9*—were selected and validated. Immune infiltration analysis revealed significant correlations between these genes and various immune cell types, particularly with *CCL27*, *ID4*, *LRP4* and *S100A9*.

**Conclusion:**

The identification of characteristic genes for cSCC provides valuable insights into its molecular mechanisms. The correlations between these genes and immune cell infiltration suggests their potential roles in tumor immunity.

## Highlights

• Utilizing GO analysis and KEGG enrichment analysis, we delved into the cellular functions and signaling pathways potentially implicated in the differentially expressed genes of cutaneous squamous cell carcinoma.
• We identified five characteristic genes in cutaneous squamous cell carcinoma through integrated analysis of differential expression gene and weighted gene co-expression network analysis.
• Our findings unveiled that four characteristic genes exhibit a strong correlation with infiltrating immune cells, suggesting their potential role in shaping the tumor immune microenvironment.

## 1 Background

Cutaneous squamous cell carcinoma (cSCC) is the second most common type of non-melanoma skin cancer (NMSC), accounting for approximately 20% of all skin cancer cases (Gong et al., 2018). In the United States, cSCC affects an estimated one million new patients annually, leading to up to 9,000 deaths each year (Karia et al., 2013). Globally, the incidence of cSCC has been rising steadily, with an annual growth rate of 10%–12% (Rogers et al., 2010). This increase poses a significant public health threat, particularly in regions with high ultraviolet exposure, where the incidence is markedly higher. (Green and Olsen, 2017). While most cases of cSCC are curable through surgical excision, approximately 4% of patients experience poor outcomes due to tumor metastasis or local recurrence (Brantsch et al., 2008). The high incidence of cSCC has resulted in a death toll comparable to that of melanoma (Fitzmaurice et al., 2019), imposing substantial physical and economic burdens on patients, a challenge likely to worsen with the aging population.

Early diagnosis and treatment of cSCC remain challenging. Early-stage cSCC often presents as skin nodules or scaly plaques, which can easily be mistaken for other benign conditions, complicating clinical diagnosis (Stratigos et al., 2020a). Although several studies have explored molecular markers for cSCC, there is currently a lack of highly specific and sensitive markers for early diagnosis, limiting their clinical application (Stratigos et al., 2020b). For metastatic cSCC, traditional treatments such as surgery and radiotherapy have shown limited efficacy in advanced stages (Que et al., 2018). Despite recent advances in targeted therapies, effective therapeutic targets for metastatic cSCC remain elusive and treatment outcomes continue to require improvement (Karia et al., 2013; Burton et al., 2016). Therefore, further investigation into the molecular mechanisms underlying cSCC and the identification of reliable biomarkers for early diagnosis and targeted therapies are essential for improving patient outcomes.

Characteristic genes involved in tumorigenesis and progression is crucial for advancing cancer research. These genes, whose abnormal expression or mutations can serve as diagnostic and prognostic biomarkers, also present potential therapeutic targets. For example, the estrogen receptor (*ER*) is a characteristic gene in breast cancer, with approximately 70%–80% of breast cancer patients being ER-positive (ER+), who typically respond well to hormone therapies such as tamoxifen (Davies et al., 2011). In the case of cSCC, mutations in genes such as *TP53*, *NOTCH1* and *CDKN2A* have been implicated in the initiation and progression of the disease (South et al., 2014). However, these mutations are not exclusive to cSCC and are observed in various other cancers. For instance, *TP53* mutations are present in approximately 42% of all cancer patients and are closely associated with tumorigenesis, progression and prognosis (Kandoth et al., 2013). Notably, *TP53* mutations are also detected in 20%–30% of cases of actinic keratosis, a precursor lesion to cSCC (Padilla et al., 2010). This suggests that the currently identified characteristic genes for cSCC lack both sensitivity and specificity. Therefore, further investigation is required to identify more specific and sensitive characteristic genes for cSCC, along with understanding their molecular mechanisms, to improve early diagnosis and targeted therapy.

High-throughput sequencing technologies, combined with weighted gene co-expression network analysis (WGCNA), has proven effective in identifying characteristic genes in cancer research. High-throughput sequencing enables comprehensive gene expression profiling, while WGCNA identifies disease-related gene modules and hub genes. For instance, in hepatocellular carcinoma, WGCNA and Least Absolute Shrinkage and Selection Operator (LASSO) algorithms have been employed to identify macrophage-related genes and further establish a prognostic model (Wang et al., 2022). While similar approaches have been applied to ovarian cancer to identify fibroblast-related genes and their roles in the tumor microenvironment (Feng et al., 2022). Despite these advances, the application of these techniques to cSCC remains unexplored. In this study, we utilized genome-wide mRNA transcriptomic data from cSCC and normal skin samples available in the Gene Expression Omnibus (GEO) database. By integrating WGCNA and LASSO algorithms, we identified characteristic genes for cSCC. These findings provide a deeper understanding of the molecular mechanisms underlying cSCC.

The tumor immune microenvironment plays a critical role in the initiation and progression of cSCC. Previous studies have demonstrated that tumor-infiltrating lymphocytes in cSCC are closely associated with immune evasion, tumor progression and patient prognosis (Saeidi et al., 2023). Additionally, tumor-associated neutrophils contribute significantly to cSCC growth and immune evasion, thereby facilitating tumor progression (Khou et al., 2020). Investigating the correlation between the expression patterns of characteristic genes and immune cells infiltration provides valuable insights into the molecular mechanisms underlying tumor growth and immune evasion. This approach also offers the potential to identify biomarkers with diagnostic, prognostic, or therapeutic relevance. In the present study, we further explored the relationship between cSCC characteristic genes and immune cell infiltration. Our findings reveal that cSCC characteristic genes are significantly correlated with the infiltration of various immune cell types. These results enhance our understanding of the immune landscape of cSCC.

## 2 Objects and methods

### 2.1 Objects

This study utilized data from patients with cutaneous squamous cell carcinoma (cSCC) obtained from the GEO database. Tumor tissue samples were classified as the “Tumor” group, while corresponding normal skin tissue samples were categorized as the “Normal” group for subsequent bioinformatics analysis.

### 2.2 Methods

#### 2.2.1 Data collection and preprocessing

Gene expression data for cSCC were retrieved from the GEO database using the search terms “Squamous cell carcinoma of skin” AND “*Homo sapiens*”. The inclusion criteria for the datasets were as follows: (1) the dataset contained genome-wide mRNA transcriptomic data, (2) the dataset included samples from both cSCC tissues and normal skin tissues and (3) datasets lacking proper annotation files were excluded. After applying these criteria, five cSCC microarray datasets were selected: GSE7553, GSE42677, GSE45164, GSE2503 and GSE117247. Among them, GSE7553, GSE42677 and GSE45164 were used as the training set for primary analysis, which included 31 cSCC tissue samples and 17 normal skin tissue samples. GSE2503 and GSE117247 were used as the test set for further validation, which included 13 cSCC tissue samples and 16 normal skin tissue samples. Descriptive statistics for the samples are shown in Supplementary Table 1. Data processing, including matrix organization, imputation, normalization and merging, was performed using the Perl and R programming languages. The limma package from the Bioconductor suite was used for data normalization and integration. Subsequently, the normalized gene expression data were used for analyses.

#### 2.2.2 Identification of differentially expressed genes (DEGs)

DEGs between the Tumor and Normal groups in the training set were identified using the limma package in R. To identify genes with significant expression changes and robust statistical significance, the screening criteria for DEGs were set as |logFC| > 2 and an adjusted P < 0.05, ensuring both biological relevance and statistical significance (Ritchie et al., 2015; Liu et al., 2019). A linear model and eBayes (empirical Bayes adjustment) were applied to enhance statistical robustness. To visualize the DEG results, heatmaps and volcano plots were generated using the pheatmap and ggplot2 R packages. These visualizations were used to highlight the most significantly altered genes between cSCC and normal skin tissue samples.

#### 2.2.3 GO and KEGG pathway enrichment analysis

To further explore the biological functions and underlying mechanisms of the identified DEGs, Gene Ontology (GO) functional enrichment analysis and Kyoto Encyclopedia of Genes and Genomes (KEGG) pathway enrichment analysis were performed. The analysis was conducted using the clusterProfiler, enrichplot and ggplot2 R packages (Yu et al., 2012). GO analysis provided insights into the biological processes, molecular functions and cellular components associated with the DEGs, while KEGG pathway analysis helped identify the key signaling pathways potentially involved in cSCC development.

#### 2.2.4 WGCNA and identification of hub genes in key module

To explore the gene interactions and their potential associations with cSCC, WGCNA was performed on the normalized gene expression data using the R package “WGCNA”. First, hierarchical clustering (hclust) was employed to construct a sample dendrogram, facilitating the detection and removal of outlier samples. The pickSoftThreshold function was then applied to determine the optimal soft-thresholding power to ensure the network’s scale-free topology. Next, gene modules were identified using the cutreeDynamic function, which employs a dynamic tree-cutting approach. The minimum module size was set to 60, and the cut height was set to 0.25. Modules with high similarity were merged. The relationship between each module and clinical phenotypic data was evaluated by calculating module-trait correlations. A module-trait heatmap was generated to highlight modules significantly associated with cSCC. Hub genes in key modules were selected based on Module Membership (MM) > 0.8 and Gene Significance (GS) > 0.5, ensuring that the identified hub genes not only play a crucial role within the module (high MM) but also exhibit a strong correlation with the studied phenotype (high GS) (Langfelder and Horvath, 2008; Ai et al., 2020). The hub genes identified in this manner were then intersected with the DEGs obtained in the previous analysis for further exploration.

#### 2.2.5 LASSO regression analysis

The intersecting genes identified in the previous step were subjected to LASSO regression analysis to further select potential characteristic genes for cSCC. A ten-fold cross-validation approach was used to determine the optimal regularization parameter lambda (λ). The value of λ corresponding to the minimum cross-validation error (λ. min = 0.0003287787) was selected as the optimal model complexity (Friedman et al., 2010). Genes with non-zero coefficients in the optimized LASSO model were identified as characteristic genes of cSCC.

#### 2.2.6 Validation of cSCC characteristic genes

To evaluate the reliability of the identified characteristic genes in cSCC, we conducted comprehensive validation through multiple approaches. Initially, the pROC package in R was used to evaluate the average achieved model performance for ten-fold cross-validation based on the intersecting genes and the characteristic genes in the training set. Subsequently, the overall performance of the model for these characteristic genes in the test set was assessed using the pROC package. Following this, comparative analysis of gene expression profiles between cSCC tumor tissues and normal was performed in both training and test sets using boxplot visualizations created with the ggpubr package.

Additionally, protein-level validation was carried out through immunohistochemical staining of three matched cSCC tumor-adjacent normal tissue pairs, confirming the translational relevance of the identified molecular signatures. Descriptive statistics for the samples are shown in Supplementary Table 2. Tissue sections underwent standard deparaffinization through xylene-ethanol series and microwave-assisted antigen retrieval in citrate buffer (pH 6.0). After cooling to room temperature and phosphate-buffered saline washing (pH 7.4), endogenous peroxidase activity was blocked with 3% H_2_O_2_. Non-specific binding was minimized through 30-min incubation with 3% bovine serum albumin (BSA). Sections were incubated overnight at 4°C with primary antibodies against ADH1B (Proteintech), CCL27 (Proteintech), ID4 (Proteintech), LRP4 (Bioss) and S100A9 (Proteintech), followed by room temperature incubation with horseradish peroxidase-conjugated secondary antibodies for 1 h. Color development was achieved using 3,3’-diaminobenzidine with hematoxylin counterstaining. Sections were subsequently dehydrated through ethanol-xylene series and mounted with neutral gum. Staining intensities were quantified as mean optical density using ImageJ software (NIH), normalized to a designated reference sample (set as 1.0 arbitrary unit), and subsequently visualized through GraphPad Prism 10 (GraphPad).

#### 2.2.7 Correlation analysis between cSCC characteristic genes and immune cell infiltration

To explore the association between characteristic genes and immune cell infiltration in cSCC, single-sample Gene Set Enrichment Analysis (ssGSEA) was used to estimate the relative abundance of 28 immune cell types across all samples. ssGSEA was performed using the normalized expression matrix and an immune gene set (GMT file), generating standardized enrichment scores that reflect the abundance of each immune cell type in each sample. Heatmaps visualizing the ssGSEA scores were constructed using the pheatmap R package. Differences in immune cell infiltration between the Tumor and Normal groups were compared using violin plots, which were created with the vioplot package, and statistical significance was assessed using the Wilcoxon rank-sum test. To further investigate the relationship between characteristic genes and immune cell infiltration, Spearman’s rank correlation coefficients (cor) were calculated using the cor. test function in R. The significance of these correlations was determined by *P*-values, and correlation heatmaps were generated using the ggplot2 package, with annotations to indicate the strength and significance of the associations.

## 3 Results

### 3.1 Identification of DEGs

**FIGURE 1:**
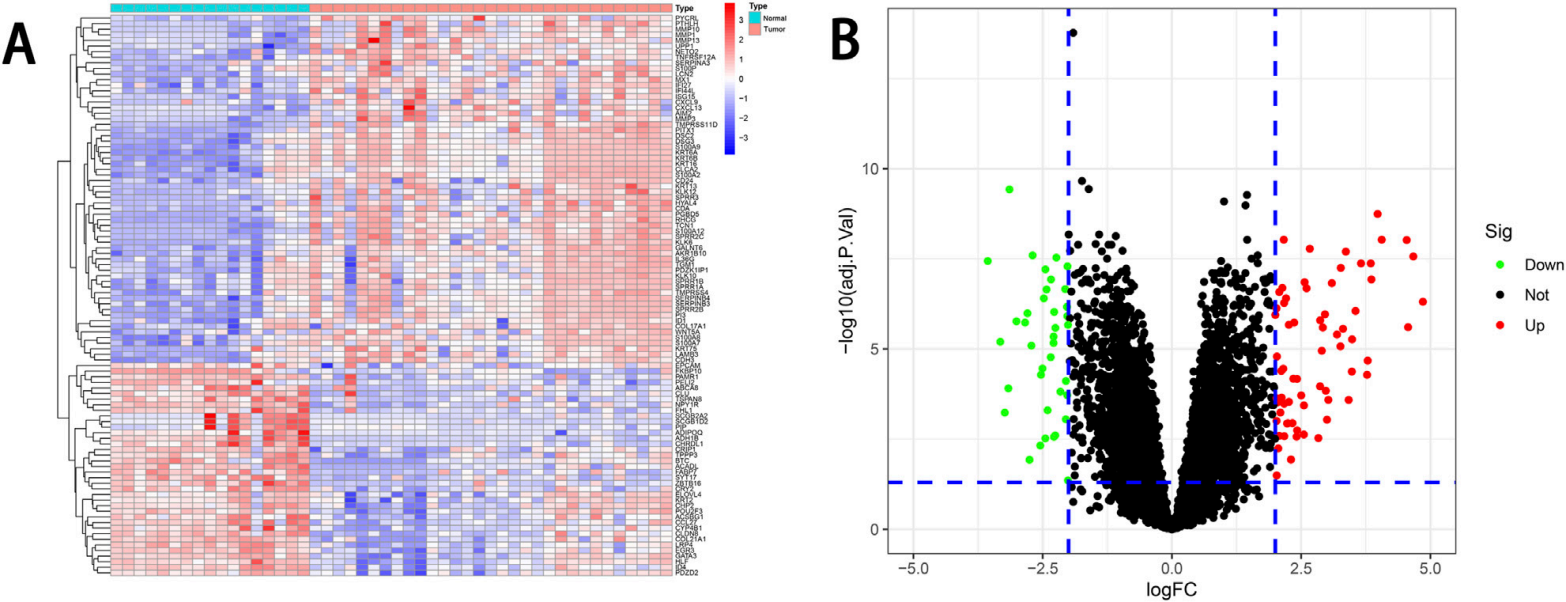
DEGs analysis. **(A)** Heatmap of DEGs. The heatmap displays the normalized expression levels of DEGs in the Normal and Tumor groups. The color scale represents the scaled expression of each gene. **(B)** Volcano plot of DEGs. The volcano plot visualizes the distribution of DEGs. Upregulated genes are represented by red dots, and downregulated genes are represented by blue dots. The threshold for the corresponding |logFC| and adjusted P-value is represented by the blue dashed line. DEGs, differentially expressed genes.

We conducted a comprehensive analysis of the preprocessed gene expression data using R, resulting in the identification of 113 DEGs. Among these, 38 genes were downregulated, and 75 genes were upregulated. The differential expression of these genes was visualized using heatmaps and volcano plots (Figure 1).

### 3.2 Functional enrichment analysis of DEGs

**FIGURE 2:**
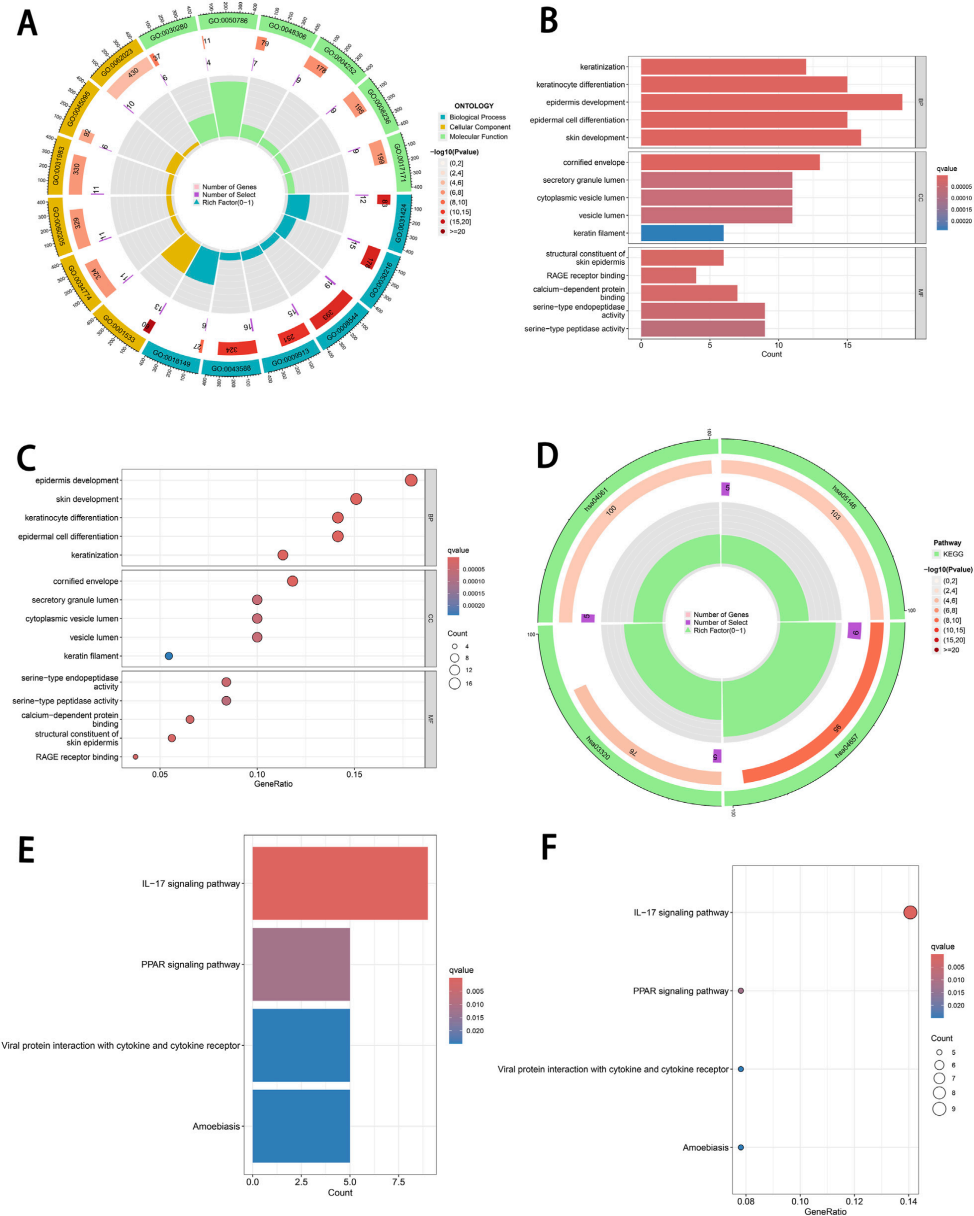
Functional enrichment analysis of DEGs. **(A)** Circular plot of GO enrichment. This plot illustrates the enriched GO terms associated with DEGs in three categories: BP, CC and MF. **(B)** Bar plot of GO enrichment. GO terms are ranked by significance (−log10(*P*-value)). **(C)** Bubble plot of GO enrichment. The size of each bubble represents the number of DEGs associated with a specific GO term, while the color gradient reflects the q-value. **(D)** Circular plot of KEGG pathway enrichment. This plot displays the KEGG pathways significantly enriched by DEGs. **(E)** Bar plot of KEGG pathway enrichment. Pathways are ranked by −log10(*P*-value), with bars showing the corresponding q-values and the number of DEGs associated with each pathway. **(F)** Bubble plot of KEGG pathway enrichment. Bubble size indicates the number of DEGs associated with each pathway, while the color gradient reflects q-value significance. DEGs, differentially expressed genes. GO, Gene Ontology. BP, biological processes. CC, cellular components. MF, molecular functions.

To further explore the biological roles and pathways of the identified DEGs, we performed GO and KEGG enrichment analyses using the ClusterProfiler package. The DEGs were categorized and analyzed in terms of biological processes, cellular components and molecular functions. In terms of biological processes, the DEGs were predominantly associated with keratinization, keratinocyte differentiation, epidermal development, epithelial cell differentiation and skin development (Figures 2A–C). These findings indicate that these genes play critical roles in skin development, keratinization, and cell differentiation. With respect to cellular components, the DEGs were enriched in the cornified envelope, secretory granule lumen, cytoplasmic vesicle lumen, vesicle lumen and keratin filaments (Figures 2A–C), underscoring their significant roles in keratinization and secretion-related processes. Regarding molecular functions, the DEGs were involved in activities such as structural constituent of skin epidermis, RAGE receptor binding, calcium-dependent protein binding, serine-type endopeptidase activity and serine-type peptidase activity (Figures 2A–C).

Furthermore, KEGG pathway analysis revealed that the DEGs were predominantly implicated in several pathways, including the IL-17 signaling pathway, PPAR signaling pathway, viral protein interaction with cytokines and cytokine receptors and amoebiasis (Figures 2D–F).

### 3.3 Identification of hub genes using WGCNA

To identify biologically significant characteristic genes, uncover potential regulatory mechanisms of cSCC, narrow the research focus, and enhance analytical efficiency, we applied WGCNA to identify gene modules and hub genes that were highly correlated with specific phenotypes.

**FIGURE 3:**
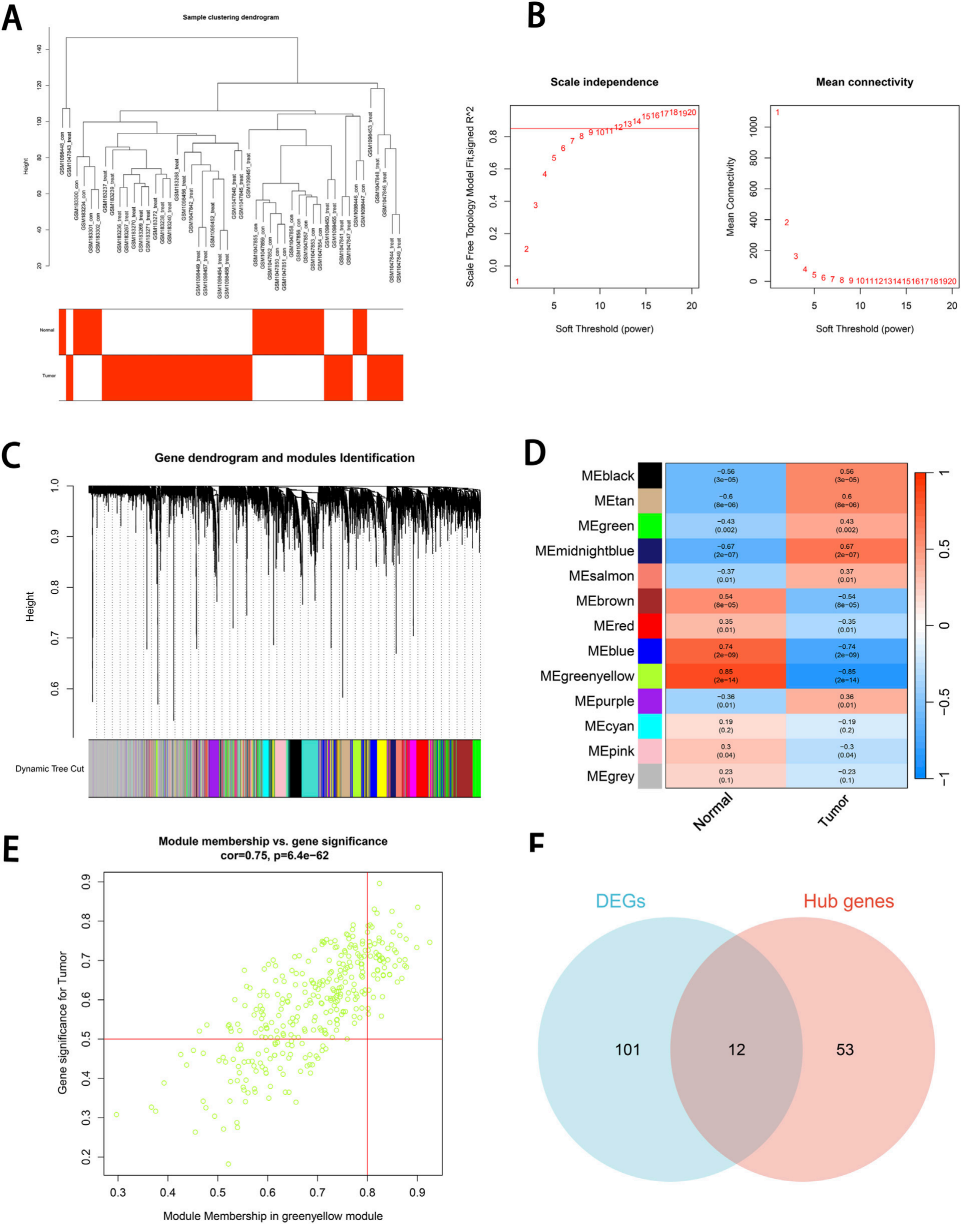
Co-expression module construction and identification of key module and hub genes using WGCNA. **(A)** Sample clustering dendrogram. Hierarchical clustering is performed based on gene expression data. **(B)** Scale Independence and mean connectivity. The scale-free topology fit index (R^2^) corresponding to soft thresholding powers (β) from 1 to 20 is shown in the left panel, and the average connectivity (k) of β values from 1 to 20 is shown in the right panel. **(C)** Gene dendrogram and modules Identification. Differentially expressed genes are clustered based on the dynamic tree cutting method, with representing different gene modules are represented by different colors. **(D)** Identification of key module. The correlation between 13 modules and two traits (Normal group and Tumor group) is shown in the figure. The correlation coefficient and significance level are indicated by the numerical values. The scaled correlations are represented by the color scale. **(E)** Identification of hub genes. A scatter plot between the module membership (MM) in the green-yellow module and the gene significance (GS) for cSCC is displayed in the figure. Genes in the upper right corner are selected as hub genes. **(F)** Venn diagram of intersecting genes. The intersecting genes between DEGs and hub genes are shown in the Venn diagram. GS, gene significance. MM, module membership.

Initially, hierarchical clustering was performed on the gene expression data to construct a sample dendrogram. No outlier samples were detected, indicating that the samples exhibited a concentrated clustering pattern and the data quality was high, providing a reliable foundation for subsequent analysis (Figure 3A). The optimal soft-thresholding power was determined to be β = 12, ensuring that the network met the scale-free topology criterion (*R* ^2^ = 0.85) while maintaining high gene connectivity. The high scale-free topology value indicated that the gene expression network adhered to the characteristics of biological networks (Figure 3B).

Subsequently, the minimum module size was set to 60, and the cut height was set to 0.25. Using the dynamic tree-cutting method, the genes were grouped into 13 modules, with each module color reflecting the similarity in gene expression patterns. These modules served as the foundation for exploring phenotype-related modules (Figure 3C). The correlation between genes within each module and the phenotypic data was calculated, and a module-trait heatmap was generated. This analysis revealed that the green-yellow module exhibited a strong positive correlation with cSCC (r = 0.85, *P* < 0.001), suggesting its potential as a key module for further analysis (Figure 3D).

Hub genes within the green-yellow module were identified based on thresholds for MM > 0.8 and GS > 0.5. We identified 65 hub genes, which not only played a crucial role within the module but also exhibited strong correlations with the phenotype of interest (Figure 3E). The hub genes were then intersected with the DEGs, and a Venn diagram was generated. This analysis revealed 12 intersecting genes that were both highly correlated within the green-yellow module and differentially expressed (Figure 3F).

### 3.4 LASSO regression analysis

**FIGURE 4:**
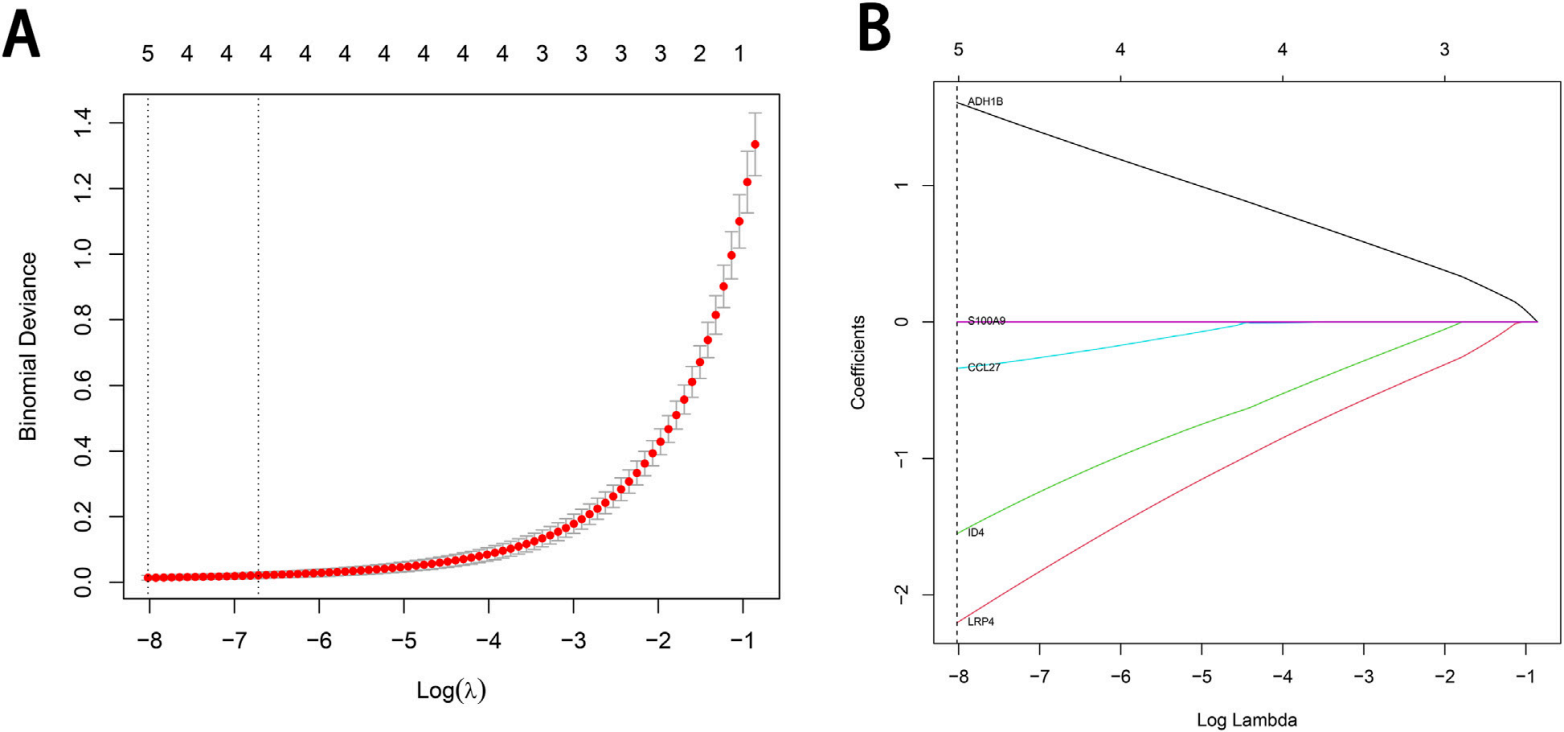
LASSO regression analysis for the selection of characteristic genes in cSCC. **(A)** The binomial deviance plot at different log(λ) values. The relationship between Log(λ) and the model fit is shown in the figure. The optimal λ value determined by cross-validation is indicated by the dashed line on the left. **(B)** The gene coefficient curve plot at different log(λ) values. The relationship between the coefficient values of different genes and Log(λ) is shown in the figure, and the figure has been marked with the selected feature genes at the optimal λ value.

To identify characteristic genes associated with cSCC while minimizing the risk of overfitting, we performed LASSO regression combined with ten-fold cross-validation on the expression levels of the 12 intersecting genes. Genes with non-zero regression coefficients were selected, and the results were visualized (Figures 4A, B). The 12 intersecting genes and their coefficients, which reflect the significance of each gene, obtained from the LASSO regression are presented in Supplementary Table 3. Ultimately, five genes—Alcohol Dehydrogenase 1B (*ADH1B*), C-C Motif Chemokine Ligand 27 (*CCL27*), Inhibitor of DNA Binding 4 (*ID4*), LDL Receptor Related Protein 4 (*LRP4*) and S100 Calcium Binding Protein A9 (*S100A9*)—were identified as characteristic genes of cSCC.

### 3.5 Validation of cSCC characteristic genes

**FIGURE 5:**
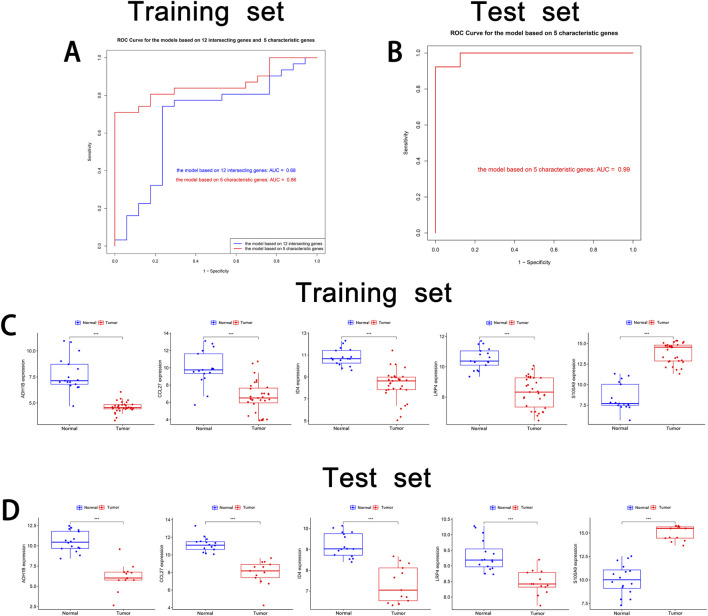
Validation of cSCC characteristic genes. **(A)** ROC curve of the training set. The ROC curve of the model based on 12 intersecting genes and five characteristic genes in the training set is shown. **(B)** ROC curve of the test set. The ROC curve of the model based on five characteristic genes in the test set is shown. **(C)** Box plot of the training set. The mRNA expression levels of five characteristic genes (*ADH1B*, *CCL27*, *ID4*, *LRP4* and *S100A9*) in the normal group and tumor group in the training set are shown. **(D)** Box plot of the test set. The mRNA expression levels of five characteristic genes in the normal group and tumor group in the test set are displayed. * indicates P < 0.05. ** indicates P < 0.01. *** indicates P < 0.001.

To validate the characteristic genes, the receiver operating characteristic (ROC) curves on the training set were used to assess the overall performance of the models based on 12 intersecting genes and five characteristic genes. The model based on 12 intersecting genes had an Area Under the Curve (AUC) value of 0.68, while the model based on five characteristic genes achieved a higher AUC of 0.86 (Figure 5A). This demonstrated that the model after combined LASSO regression offers superior predictive performance. Additional performance metrics, such as sensitivity and specificity are provided in Supplementary Table 4. Subsequent external validation on the test set revealed exceptional generalizability, demonstrating robust discriminative capacity with an AUC of 0.99 (Figure 5B).

**FIGURE 6:**
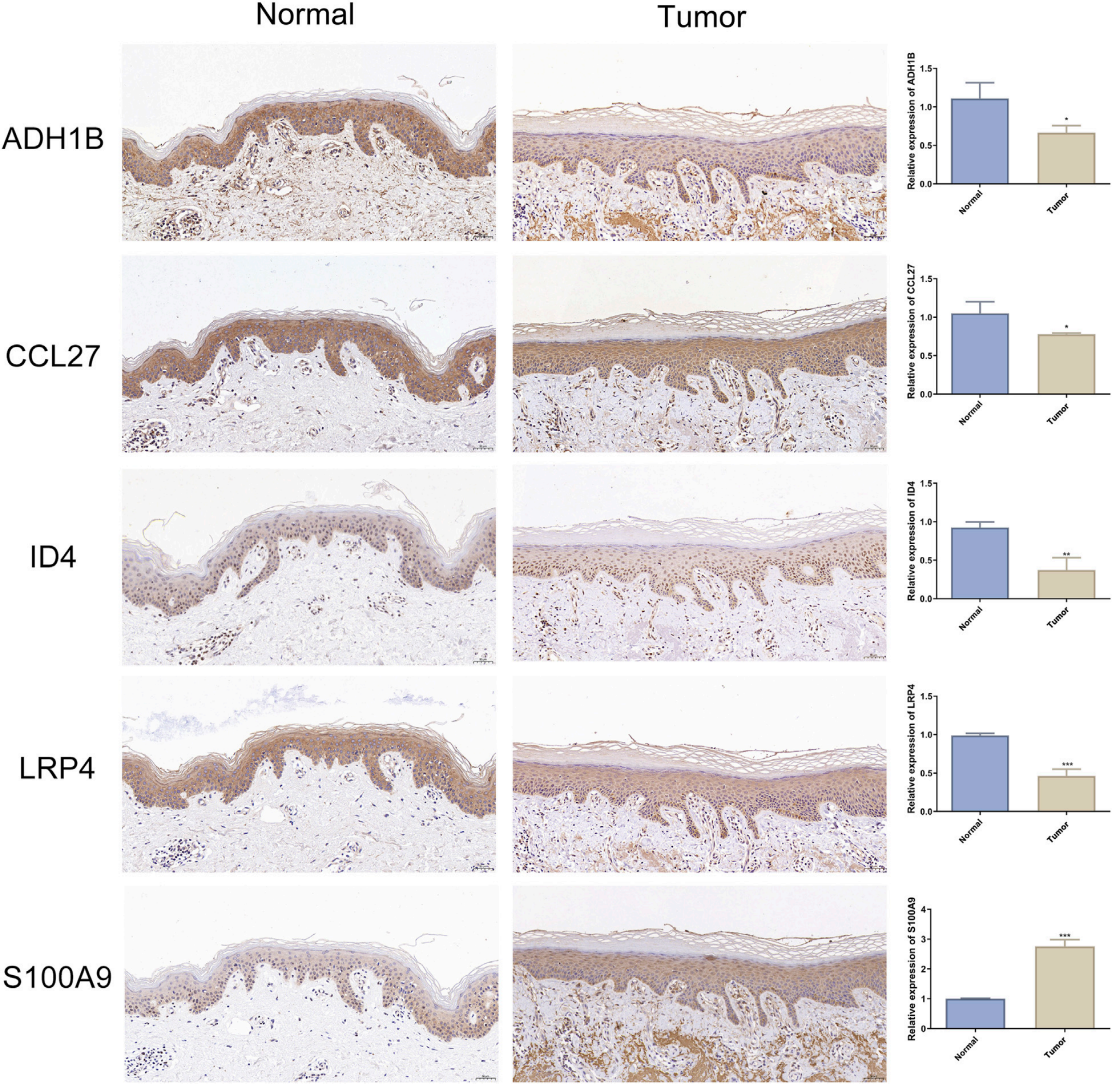
Expression of cSCC characteristic genes in immunohistochemistry. Representative immunohistochemical images of ADH1B, CCL27, ID4, LRP4 and S100A9 expression in adjacent normal skin (left panel) and cSCC tumor (middle panel). Nuclei were counterstained with hematoxylin (blue), with positive signals shown by 3,3’-diaminobenzidine deposition (brown). Scale bars: 50 μm. Quantitative analysis of normalized expression levels (right panel). Data expressed as Mean ± SD. n = 3. * indicates P < 0.05. ** indicates P < 0.01. *** indicates P < 0.001.

mRNA expression analysis, visualized using boxplots generated by ggpubr, revealed consistent expression patterns between the training and test sets for all characteristic genes, with statistically significant differential expression (Figures 5C, D). This finding confirmed the stable identification of these genes across distinct patient cohorts. Notably, to corroborate the transcriptional findings at protein level, we conducted comparative immunohistochemical analysis between cSCC lesions and matched normal skin tissues. The protein expression profiles demonstrated distinct differential patterns that mirrored the mRNA expression trends (Figure 6). Quantitative evaluation revealed consistent downregulation of ADH1B, CCL27, ID4 and LRP4 in tumor tissues compared to normal counterparts. Conversely, S100A9 exhibited marked upregulation in tumor. This multi-level concordance between transcriptional and translational data strongly corroborates the biological relevance of these characteristic genes in cSCC development.

### 3.6 Correlation analysis between cSCC characteristic genes and infiltrating immune cells

Although the five characteristic genes were identified, their exact roles in cSCC tumorigenesis and progression remain unclear. Given the established connection between the immune microenvironment and cSCC development (Saeidi et al., 2023), and the functional enrichment analysis indicating the involvement of these genes in immune-related pathways (e.g., *S100A9* in the IL-17 signaling pathway as shown in Supplementary Table 5), we further explored the relationship between these genes and the infiltration of immune cells.

**FIGURE 7:**
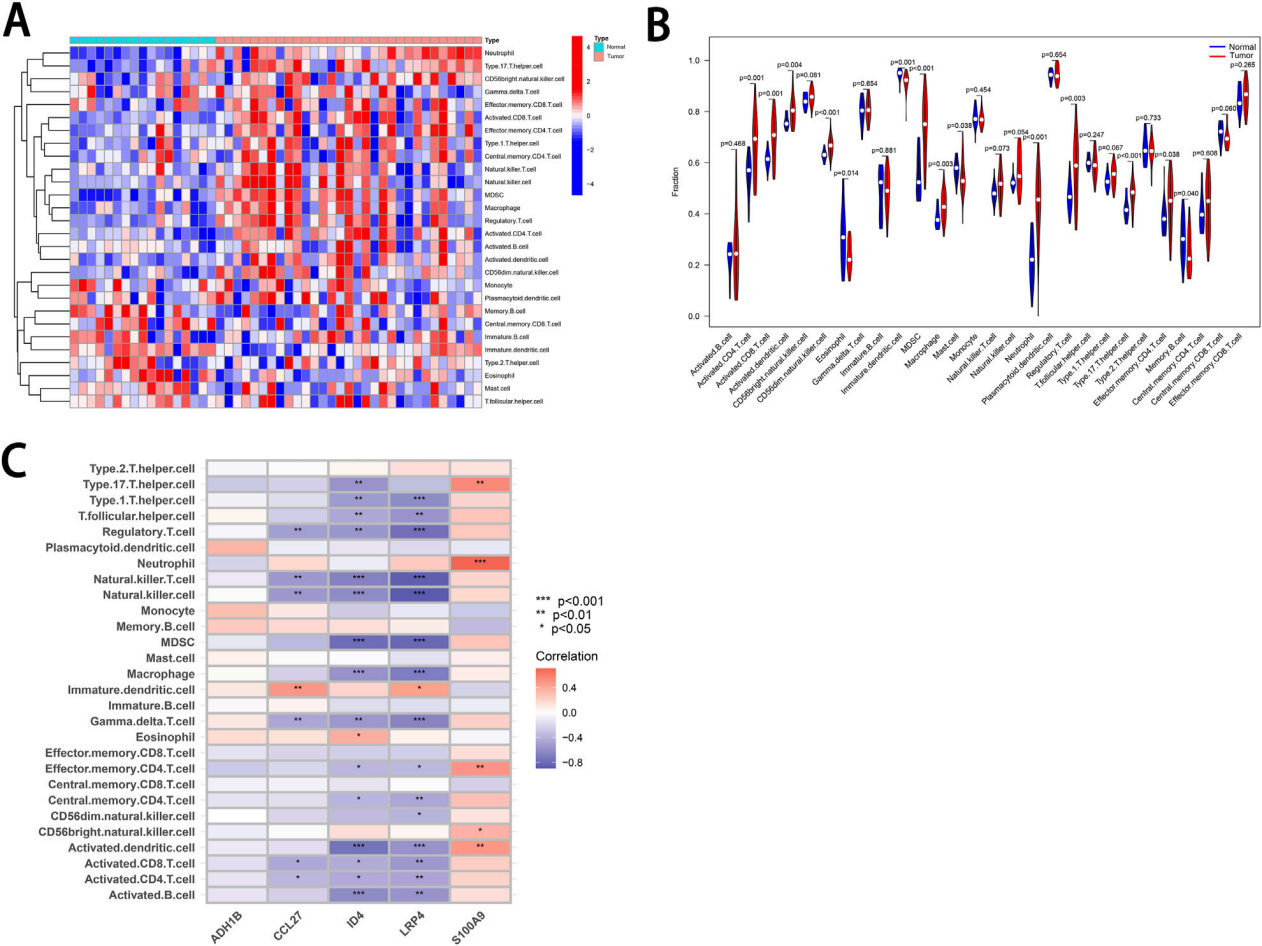
Correlation of characteristic genes with infiltrating immune cells in cSCC. **(A)** Heatmap. This heatmap illustrates the relative abundance of 28 infiltrating immune cell types in the tumor and normal groups. The color scale represents the degree of infiltration for each cell type. **(B)** Violin Plot. This plot compares the differences in immune cell infiltration between the tumor and normal groups. *P*-values are provided to assess statistical significance. **(C)** Correlation Heatmap. The picture depicts the relationships between five characteristic genes and various infiltrating immune cell types. The color scale represents positive correlations in red and negative correlations in blue. * indicates P < 0.05. ** indicates P < 0.01. *** indicates P < 0.001.

Using the ssGSEA method, we analyzed the infiltration of 28 immune cell types in the Tumor and Normal groups. The results revealed statistically significant differences in the infiltration of 14 immune cell types (Supplementary Table 6). Among these, 10 immune cell types exhibited higher levels of infiltration in the Tumor group, while five showed significantly lower levels in the Tumor group compared to the Normal group (Figures 7A, B).

Furthermore, we performed a correlation analysis between the cSCC characteristic genes and the abundance of infiltrating immune cells (Figure 7C). The analysis revealed that four of the characteristic genes—*CCL27*, *ID4*, *LRP4* and *S100A9*—were significantly associated with various types of infiltrating immune cells. For instance, *S100A9* expression was positively correlated with the abundance of Type 17 T helper cells, neutrophils, effector memory CD4 T-cell, CD56 bright natural killer cells and activated dendritic cells. The correlations between the five characteristic genes and various immune cell types, along with their corresponding *P*-values, are provided in Supplementary Table 7. These results suggest that the characteristic genes *CCL27*, *ID4*, *LRP4* and *S100A9* may play a role in modulating the immune microenvironment of cSCC.

## 4 Discussion

cSCC, a major subtype of NMSC, is characterized by its potential for local invasion and distant metastasis. Although the overall metastatic rate is relatively low, with approximately 4% of patients developing lymph node metastasis and 1.5% succumbing to the disease (Karia et al., 2013), advanced cSCC remains a significant clinical challenge. Early-stage cSCC can be effectively treated with current therapies, but management of advanced cases continues to pose considerable difficulties. Understanding the characteristic genes involved in cSCC pathogenesis is critical for elucidating the mechanisms underlying its development and progression, and for advancing diagnostic and therapeutic strategies. However, research into the molecular profiles of cSCC remains limited, and systematic investigations to identify and explore characteristic genes are urgently needed.

In the present study, we identified five characteristic genes from three cSCC datasets (GSE45164, GSE42677 and GSE7553) for the first time. While the original study of GSE42677 also identified *S100A9* as a DEG, our combined approach, which includes WGCNA, LASSO regression analysis and immunohistochemical staining, further validates that *S100A9*, along with the other four genes, collectively serves as a set of characteristic genes for cSCC. Due to significant differences in research objectives, analytical methods, sample sizes and sample compositions, the conclusions drawn in our study exhibit notable disparities from those of the original studies.

Among these characteristic genes, *ADH1B*, *CCL27*, *ID4* and *LRP4* were found to be significantly downregulated in cSCC (Figure 5C). Previous studies have highlighted the roles of these genes in the tumorigenesis and progression of other cancers, suggesting that their altered expression may be crucial for the development of cSCC. For instance, polymorphisms in the *ADH1B* gene, such as rs1229984, have been shown to correlate with a reduced risk of head and neck cancer (Imani et al., 2024). Additionally, *ADH1B* expression is notably reduced in hepatocellular carcinoma (HCC), with low expression levels associated with poor prognosis (Liu et al., 2020). Although our study does not provide direct evidence linking *ADH1B* to cSCC prognosis, its downregulation suggests that it may be involved in disease progression, warranting further investigation in clinical cohorts. *CCL27*, a chemokine involved in immune cell recruitment and lymphangiogenesis, has been shown to be upregulated in several cancers, including breast cancer and melanoma, where it contributes to tumor metastasis by promoting lymphatic spread (Karnezis et al., 2019). However, this study indicates that *CCL27* is downregulated in cSCC. Given that cSCC exhibits significantly lower lymph node metastatic potential compared to breast cancer and melanoma, this finding suggests that *CCL27* may play a role in the differential lymphatic metastasis capabilities among various cancers. Further research is needed to elucidate the molecular and biological functions of *CCL27* in tumor metastasis. The expression of ID4 has been reported to be suppressed by UVB irradiation via DNA methylation, which in turn promotes cell proliferation, migration and invasion, contributing to tumor formation in mouse models of skin cancer (Li et al., 2020). In non-small cell lung cancer (NSCLC), *ID4* upregulation is associated with improved prognosis, suggesting its potential role as a tumor suppressor (Rodón et al., 2019). Similarly, ID4 was found to be downregulated in cSCC in our study. It remains unclear whether this downregulation contributes to cSCC progression, and further experimental validation is needed to explore its potential role. LRP4, a member of the low-density lipoprotein receptor family, has been implicated in various cancers. In NSCLC, LRP4 downregulation promotes tumorigenesis and progression by modulating the Wnt/β-catenin signaling pathway (Zhang et al., 2024). In contrast, *LRP4* is upregulated in papillary thyroid carcinoma, where it promotes cell proliferation, migration and invasion (Zhou et al., 2018). This contradictory expression pattern suggests that LRP4 may have context-dependent roles in different cancer types, potentially influenced by the tumor microenvironment. However, the prognostic significance of *LRP4* in cSCC remains to be determined, and further studies are needed to explore its functional role. In summary, our findings reveal that four of the identified characteristic genes (ADH1B, CCL27, ID4 and LRP4) are consistently downregulated in cSCC, and prior studies suggest their involvement in tumor proliferation, migration and invasion. However, the specific roles of these genes in cSCC require further clinical and experimental validation.

In addition to the four downregulated characteristic genes mentioned, this study also confirmed the upregulation of *S100A9* in cSCC (Figure 5C). *S100A9* is a member of the S100 protein family, which binds calcium and plays a critical role in the recruitment and adhesion of immune cells such as neutrophils and monocytes, especially in inflammatory responses and inflammation-associated diseases (Wang et al., 2018). Previous studies have demonstrated that *S100A9* is upregulated in various cancers and is closely associated with tumor initiation, progression and prognosis (Acharyya et al., 2012; Eisenblaetter et al., 2017). In cSCC, elevated *S100A9* expression has been found to bind to Receptor for Advanced Glycation Endproducts on myeloid-derived suppressor cells (MDSCs), activating NF-κB signaling. This interaction promotes the recruitment of MDSCs to the tumor site, which suppresses the immune system’s anti-tumor response, thereby facilitating tumor growth and metastasis (Markowitz and Carson, 2013). Our study also observed a potential positive correlation between *S100A9* and MDSC infiltration (Figure 7C). While the p-value was not sufficient to confirm statistical significance, the results partially support previous findings. Interestingly, downregulation of *S100A9* expression in cSCC has been shown to inhibit tumor proliferation and migration (Zhang et al., 2021). Furthermore, IL-6 has been clinically associated with poor prognosis in cSCC (Mallardo et al., 2023), thereby underscoring the critical involvement of inflammatory molecules in tumor progression. Future investigations into how these inflammatory mediators collaboratively orchestrate tumor development through synergistic molecular networks will be of paramount importance for advancing therapeutic strategies.

Notably, the intricate tumor immune microenvironment involves not only MDSC-mediated immunosuppression but also dynamic equilibrium among immune cell populations. Our study revealed concurrent infiltration of functionally antagonistic CD8^+^ cytotoxic T-cell and immunosuppressive Tregs within tumor tissues, a paradoxical coexistence that has been previously documented in renal clear cell carcinoma and lung adenocarcinoma (Pan et al., 2020; Meng et al., 2024). Current research demonstrates functional heterogeneity in tumor-infiltrating CD8^+^ T-cell, where many exhibit non-reactive “bystander” phenotypes that challenge the prognostic utility of quantitative assessments alone (Simoni et al., 2018). Moreover, Treg cell-mediated immunosuppression further impairs the functional state of CD8^+^ T-cell (Sakaguchi et al., 2008). These findings collectively suggest that effective immunotherapeutic strategies should adopt dual targeting approaches: enhancing effector T-cell functionality while selectively inhibiting Treg-mediated immunosuppression to achieve durable antitumor responses.

Furthermore, in this study, we established correlations between the cSCC characteristic genes and various infiltrating immune cell populations through bioinformatics analysis (Figure 7C). These findings suggest that these genes may play a role in shaping the cSCC immune microenvironment and promoting immune evasion, a hypothesis supported by previous studies. For instance, *S100A9* is highly expressed in neutrophils and monocytes and acts as a promoter by regulating tumor metabolism and the immune microenvironment, thereby facilitating tumor growth and metastasis (Chen et al., 2023). Further research has revealed that tumor-associated neutrophils can suppress CD8^+^ T-cell function and express Programmed Death-Ligand 1, contributing to immune evasion and promoting cSCC progression (Khou et al., 2020). Our study reaffirms the positive correlation between high S100A9 expression and increased neutrophil infiltration in cSCC, consistent with previous reports. While there is substantial evidence supporting the involvement of *S100A9* in the tumor immune microenvironment and immune evasion, the roles of *CCL27*, *ID4* and *LRP4*, genes that also showed strong correlations with infiltrating immune cells, remain inadequately explored. Future research is needed to clarify whether these genes influence the tumor immune environment and to elucidate their specific mechanisms. Such studies will deepen our understanding of cSCC and could uncover novel targets for immunotherapy.

However, this study has several limitations. First, the available datasets that met the analysis criteria were limited, resulting in a relatively small sample size that may not fully capture the heterogeneity of cSCC. Second, although multiple screening methods were employed, the stability and generalizability of the identified characteristic genes require validation in larger, multi-center cohorts. Additionally, the prognostic significance of these genes in cSCC has not been systematically evaluated, which should be addressed in future studies. Future research should focus on expanding sample sizes, incorporating diverse data sources, conducting functional validation experiments, integrating multi-omics data, and performing prospective clinical studies to improve the representativeness and clinical applicability of the findings.

In conclusion, this study employed bioinformatics techniques, including WGCNA, to identify five characteristic genes associated with cSCC: *ADH1B*, *CCL27*, *ID4*, *LRP4* and *S100A9*. Additionally, we demonstrated that *CCL27*, *ID4*, *LRP4* and *S100A9* are correlated with the infiltration of various immune cell types in cSCC. Further investigation into the roles and mechanisms of these characteristic genes in cSCC formation, progression, migration and immune evasion will enhance our understanding of the disease and may provide a foundation for future diagnostic biomarkers and therapeutic targets.

## Data Availability

The datasets presented in this study can be found in online repositories. The names of the repository/repositories and accession number(s) can be found below: https://www.ncbi.nlm.nih.gov/geo/, GSE42677; https://www.ncbi.nlm.nih.gov/geo/, GSE45164; https://www.ncbi.nlm.nih.gov/geo/, GSE7553.
